# Effect of soybean oligopeptide on the growth and metabolism of *Lactobacillus acidophilus* JCM 1132

**DOI:** 10.1039/d0ra01632b

**Published:** 2020-04-29

**Authors:** Wenhui Li, Yinxiao Zhang, He Li, Chi Zhang, Jian Zhang, Jalal Uddin, Xinqi Liu

**Affiliations:** Beijing Advanced Innovation Center for Food Nutrition and Human Health, Beijing Engineering and Technology Research Center of Food Additives, Beijing Technology and Business University Beijing 100048 China lihe@btbu.edu.cn liuxinqi@btbu.edu.cn

## Abstract

Soybean protein (Pro) and soybean oligopeptide (Pep) were subjected to simulated digestion *in vitro* to study the effect of Pep on the growth and metabolism of *Lactobacillus acidophilus* JCM 1132. First, the molecular weight distribution differences of samples before and after digestion were compared, and the samples were used to replace the nitrogen source components in the culture media. Then, the viable cell numbers, lactic acid and acetic acid content, differential metabolites, and metabolic pathways during the culturing process were measured. Results showed that the digested soybean oligopeptide (dPep) was less efficient than MRS medium in promoting the growth, but by increasing the content of the intermediates during the tricarboxylic acid (TCA) cycle, its metabolic capacity was significantly improved. Besides, due to the low molecular weight of dPep, it can be better transported and utilized. And dPep significantly strengthened the amino acid metabolism and weakened the glycerol phospholipid metabolism, so the ability of dPep in promoting the growth and metabolism of *Lactobacillus acidophilus* JCM 1132 is higher than the digested soybean protein (dPro).

## Abstract 2

Exploring the effect of soybean oligopeptide on the growth and metabolism of *Lactobacillus acidophilus* JCM 1132 by metabolomics.

## Graphical abstract

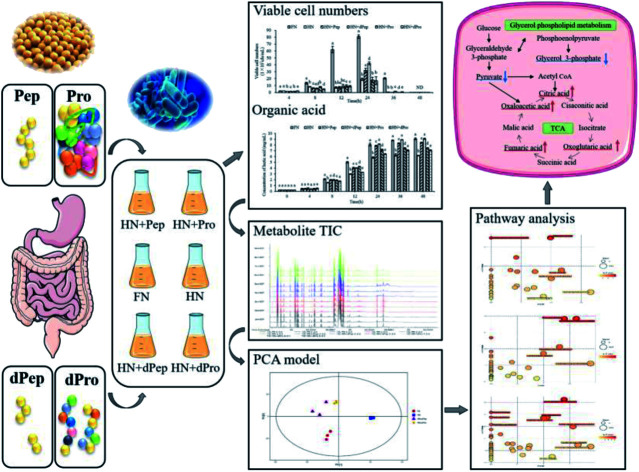

## 1. Introduction

Although Pro is a high-quality, nutritionally balanced plant protein displaying an excellent amino acid composition, it has the disadvantage of poor solubility and high allergenicity.^1–3^ Pep consists of protein hydrolysates obtained by proteolytic hydrolysis and purification of Pro as raw materials, generally consisting of 3–6 amino acids, while most of the peptides have a molecular weight below 1000 Da.^4^ Pep not only exhibits the same advantages as Pro but also displays excellent solubility, high stability, easy absorption and low antigenicity, which are characteristics absent from Pro. In addition, it presents a variety of biological activities responsible for lowering cholesterol, acting as antioxidants, and improving hypertension.^5–8^

At present, most of the research on prebiotics focuses on carbon sources such as fructooligosaccharides and xylooligosaccharides and often ignore the influence of nitrogen sources on probiotics.^9–11^ Nitrogen sources are essential for the growth of probiotics, and an insufficient nitrogen source leads to the decelerated growth and metabolism of the probiotics even when the high-dose carbon source is supplemented.^12^ The nitrogen sources in the daily diet mainly consist of proteins and peptides, while proteins, peptides, and amino acids mainly represent those that reach the intestine and directly acts on the intestinal flora after digestion by the human body.^13,14^

Probiotics are mainly composed of *Lactobacillus* and *Bifidobacterium*. The latest definition presents probiotics as live microorganisms which, when administered in adequate amounts, confer a health benefit on the host.^15^ The proliferation of probiotics and the production of their metabolites can effectively regulate the structure of intestinal flora, enhance immunity, promote mental health, reduce blood pressure, and treat liver diseases.^16–18^*Lactobacillus acidophilus*, which belongs to *Lactobacillus*, is one of the vital intestinal probiotics and is closely related to the health of the host. Furthermore, it serves many functions, such as regulating the intestinal epithelial barrier, inhibiting pathogenic bacteria, acting as an anti-inflammatory, and has seen increasing application in the food, medicine and foraging fields.^19–23^ However, the cell-envelope proteinase (CEP) secretion ability of *Lactobacillus acidophilus* is weak, rendering the utilization ability of macromolecular protein inferior.^24,25^ However, the peptide supplement can be directly transported into the *Lactobacillus acidophilus* cells and hydrolyzed by peptidase, while the growth and metabolism capacity of *Lactobacillus acidophilus* can also be improved.^26^

Most of the research involving the impact of peptides on *Lactobacillus acidophilus* concentrates on the extraction of peptides from different sources and their effect on the growth of probiotics, such as the extraction and characterization of peptides from cheese, the preparation of protein hydrolysates from poultry processing residues, the preparation of egg white hydrolysates and their effect on the growth of *Lactobacillus* and *Bifidobacterium*, respectively.^12,27,28^ Regarding the raw soybean materials, some studies have confirmed that Pro hydrolysate can promote probiotics such as *Lactobacillus acidophilus*, but none of them involve the effect of dPro and dPep on the growth and metabolism of *Lactobacillus acidophilus*, and no study explores the various effects of Pep on the differential metabolites and metabolic pathways of *Lactobacillus acidophilus* compared with Pro and MRS medium.^29^

In this experiment, Pep and Pro are digested *in vitro*, and the molecular weight distribution of the sample is detected before and after digestion, after which *Lactobacillus acidophilus* JCM 1132 is cultured further with a nitrogen source replacement. Then, the effect of Pep on its growth and metabolism is compared with that of Pro and MRS medium. Furthermore, the differences of metabolites are compared, and the pathway of Pep promoting the growth and metabolism of *Lactobacillus acidophilus* JCM 1132 is studied. Therefore, this study aims to explore the potential mechanism of Pep as a nitrogen source for probiotics, to develop a new perspective for the study of intestinal flora and perform further research.

## 2. Materials and methods

### 2.1 Materials and microorganisms

Pro and Pep were purchased from the Nutrily Biotechnology, Ltd. (Anyang, Henan, China). *Lactobacillus acidophilus* JCM 1132 was purchased from the China General Microbiological Culture Collection Center (Beijing, China). Pepsin and trypsin were purchased from the Novozymes (Beijing, China). Bicinchoninic acid (BCA) protein assay kit, acrylamide, glycine, sodium dodecyl sulfate (SDS), Tris, urea and loading buffer were purchased from Solarbio (Beijing, China). Ammonium persulfate and tetramethylethylenediamine (TEMED) were purchased from Amersco (Framingham, USA). Coomassie Brilliant Blue R-250 was purchased from Bio-Rad (Richmond, CA, USA). MRS broth and MRS agar were purchased from Aobox Biotechnology, Ltd. (Beijing, China). Bis trifluoroacetamide (BSTFA), fatty acid methyl ester (FAME), methoxy amination hydrochloride, trimethyl chlorosilane (TMCS), standards of lactic acid, acetic acid, glycine, Gly-Gly-Gly, bacitracin and insulin were purchased from Sigma-Aldrich (St. Louis, MO, USA). 2-Chloro-l-phenylalanine was purchased from Hengbai Biological Technology, Ltd. (Shanghai, China). High performance liquid chromatography (HPLC) grades of acetonitrile, methanol, chloroform, pyridine and trifluoroacetic acid (TFA) were purchased from Fisher Scientific (Ottawa, ON, Canada).

### 2.2 The digestion of Pep and Pro *in vitro*

**Fig. 1:**
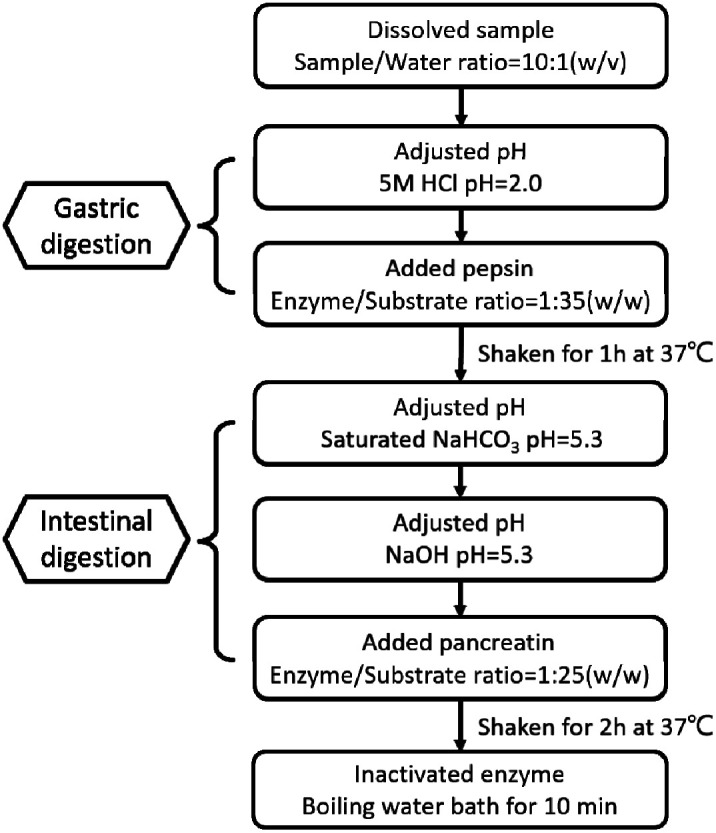
Simulated digestion process of Pep and Pro *in vitro*. Pep = soybean oligopeptide, Pro = soybean protein.

The effect of Pep and Pro on *Lactobacillus acidophilus* JCM 1132 after reaching the intestine, by digesting the two samples *in vitro*, referring to the standardized static *in vitro* digestion method that is applied to food with some modifications. The specific method is shown in Fig. 1.^30,31^ After termination of the digestion process, the samples were stored at −40 °C and freeze-dried for 48 h to obtain the powder of the dPep and the dPro.

### 2.3 Molecular weight distribution analysis

The molecular weight distribution of Pro was determined with sodium dodecyl sulfate-polyacrylamide gel electrophoresis (SDS-PAGE). First, 30% acrylamide solution, 1.5 M Tris–HCl, 1 M Tris–HCl, 10% SDS solution, and 10% ammonium persulfate solution were prepared and mixed in proportion with the solutions mentioned above, after which a 12% separation gel and a 5% concentration gel were prepared. A BCA protein assay kit was used to measure the protein concentration of Pro and dPro. After adjusting the amount of protein to be loaded to about 10 μg, it was mixed with the loading buffer (v/v = 4 : 1), after which the protein in the samples was denatured in a 95 °C water bath for 10 min. Then, 10 μL was applied to each well, and the voltage was adjusted to 80 V for electrophoresis. This process was followed by using Coomassie brilliant blue R-250 staining to show the protein bands, after which the image was analyzed by gel imaging system (Bio-Rad, Richmond CA, USA) after decolorization.^32,33^

Due to the small molecular weight of Pep and to further determine its molecular weight distribution, an Agilent 1260 HPLC-DAD system was performed. The powder of the standards and the samples were prepared as a solution of 1 mg mL^−1^, which were filtered through 0.22 μm microfiltration membrane, and then analyzed using a TSKgel G2000SW_XL_ chromatographic column (300 mm × 7.8 mm) (TOSOH, Japan). The mobile phase consisted of acetonitrile, water, and TFA = 45 : 55 : 0.1, with a sample volume of 10 μL, and a flow rate of 0.5 mL min^−1^, while the detection was performed at a wavelength of 220 nm.^34^

### 2.4 The preparation of the culture medium and the determination of viable cell numbers

Here, the differences between the impact of Pro and Pep on the growth capacity of *Lactobacillus acidophilus* JCM 1132 were evaluated. First, the nitrogen content of three components (peptone, beef extract and yeast extract) in the MRS medium and Pep, dPep, Pro, dPro were measured using the Kjeltec 8400 system (FOSS, Denmark) (Table 1). Then, half of the nitrogen source components in the MRS medium were reduced, while the Pep, dPep, Pro, dPro were simultaneously supplied with the same nitrogen content. The experiment was divided into six groups: MRS medium group (FN), MRS medium without half nitrogen source group (HN), HN supplemented Pep group (HN + Pep), HN supplemented dPep group (HN + dPep), HN supplemented Pro group (HN + Pro), HN supplemented dPro group (HN + dPro). Except for the HN group, the nitrogen content of other groups remained the same. The specific groups and the preparation of the culture medium are shown in Table 2.

**Table 1:** Nitrogen content of medium composition and samples^a^

Ingredient/Sample	Nitrogen content (mg N g^−1^)
Peptone	157.30 ± 1.15
Beef extract	134.66 ± 1.41
Yeast extract	108.40 ± 0.95
Pep	143.58 ± 1.00
dPep	101.40 ± 0.97
Pro	134.70 ± 1.63
dPro	109.30 ± 1.58

a Pep = soybean oligopeptide, dPep = digested soybean oligopeptide, Pro = soybean protein, dPro = digested soybean protein. Data are shown as mean ± standard deviation.

**Table 2:** Composition of different nitrogen source media^a^

Ingredient	Different nitrogen source media					
	FN (g L^−1^)	HN (g L^−1^)	HN + Pep (g L^−1^)	HN + dPep (g L^−1^)	HN + Pro (g L^−1^)	HN + dPro (g L^−1^)
Peptone	10	5	5	5	5	5
Beef extract	5	2.5	2.5	2.5	2.5	2.5
Yeast extract	4	2	2	2	2	2
Pep	—	—	9.33	—	—	—
dPep	—	—	—	13.21	—	—
Pro	—	—	—	—	9.95	—
dPro	—	—	—	—	—	12.26
Glucose	20	20	20	20	20	20
Sodium acetate	5	5	5	5	5	5
Dipotassium phosphate	2	2	2	2	2	2
Triammonium citrate	2	2	2	2	2	2
Magnesium sulfate	0.2	0.2	0.2	0.2	0.2	0.2
Manganese sulfate	0.05	0.05	0.05	0.05	0.05	0.05
Polysorbate 80	1	1	1	1	1	1

a Pep = soybean oligopeptide, dPep = digested soybean oligopeptide, Pro = soybean protein, dPro = digested soybean protein.

After the preparation was completed, it was autoclaved at 121 °C for 15 min, and cooled to room temperature, after which 2% (v/v) *Lactobacillus acidophilus* JCM 1132 was added to the media of each group. Furthermore, to control the sterility of these media, a control without inoculum was always included to prove the absence of growth. After inoculation, six media were stationary cultured at 37 °C for 48 h and sampled at seven time points of 0 h, 4 h, 8 h, 12 h, 24 h, 36 h, and 48 h. The cultures at each time point were then diluted in 10-fold serial dilution independently, a 10^−6^ diluted solution was selected and plated on MRS agar medium at 37 °C for 48 h.^35,36^ Each assay was performed in triplicate. All the procedures mentioned above were performed on a clean bench.

### 2.5 The determination of pH and organic acids

*Lactobacillus acidophilus* JCM 1132 was cultured in six media and sampled at seven time points of 0 h, 4 h, 8 h, 12 h, 24 h, 36 h, and 48 h to evaluate the differences in its metabolic capacity when exposed to Pep and other samples, respectively. One part of the sample was used to measure the pH value, while the remaining part was for organic acid content measurement and centrifugation at 4 °C and 12 000 rpm for 10 min. Then, the supernatant was collected and quenched with liquid nitrogen, after which it was stored at −80 °C.

The method used for the detection of organic acids is based on some research with some modifications.^37,38^ The lactic acid and acetic acid standards were formulated into different concentrations, after which the standard solution and the bacterial culture solution collected at each time point were passed through a 0.22 μm microfiltration membrane. Finally, these samples were analyzed on an ion-exchange Aminex HPX-87H Column (300 mm × 7.8 mm) (Bio-Rad, Richmond CA, USA) in the HPLC-DAD system. The mobile phase consisted of 13 mM sulfuric acid with an injection volume of 10 μL, a flow rate of 0.8 mL min^−1^, a temperature of 65 °C, and a detection wavelength of 220 nm.

### 2.6 Metabolite extraction

The different effects of Pep and other samples on the metabolic capacity of *Lactobacillus acidophilus* JCM 1132 was further explored by extracting the metabolites from the bacterial culture solution using the following procedure. The 24 h bacterial culture (300 μL) was mixed with methanol (300 μL) and 2-chloro-l-phenylalanine (10 μL), after which it was vortexed and treated ultrasonically for 10 min. Then, it was centrifuged at 4 °C and 12 000 rpm for 15 min. After this process, 50 μL of the supernatant was collected from each sample and pooled as a quality control (QC) sample, the extract was dried in a vacuum concentrator. Then, 60 μL methoxy amination hydrochloride (20 mg mL^−1^ in pyridine) was added and incubated at 80 °C for 30 min. Subsequently, 80 μL of the BSTFA regent (1% TMCS, v/v) was added to the sample aliquots, and incubated at 70 °C for 1.5 h, followed by the addition of 5 μL FAME (in chloroform) to the QC sample while the mixture was cooling to the room temperature.^39^

### 2.7 Metabolite detection

GC-TOF-MS analysis was performed using an Agilent 7890 gas chromatograph system coupled with a Pegasus HT time-of-flight mass spectrometer. The system utilized a DB-5MS capillary column coated with 5% diphenyl, cross-linked with 95% dimethylpolysiloxane (30 m × 0.25 mm, 0.25 μm film thickness) (J&W Scientific, Folsom, CA, USA). A 1 μL aliquot of the analyte was injected in splitless mode with helium was as the carrier gas, a front inlet purge flow of 3 mL min^−1^, and a gas flow rate through the column of 1 mL min^−1^. The initial temperature was maintained at 50 °C for 1 min, then raised to 310 °C at a rate of 10 °C min^−1^ where it was maintained for 8 min. The injection, transfer line, and ion source temperatures were 280 °C, 280 °C, and 250 °C, respectively. The energy was −70 eV in electron impact mode. The mass spectrometry data were acquired in full-scan mode with an *m*/*z* range of 50–500 at a rate of 12.5 spectra per second after a solvent delay of 6.33 min. Chroma TOF 4.3X software from the LECO Corporation and the LECO-Fiehn Rtx5 database were used for the raw peak exacting, data baseline filtering, and calibration of the baseline, peak alignment, deconvolution analysis, peak identification and integration of the peak area. Both the mass spectrum match and retention index match were considered during metabolite identification. The peaks detected in <50% of the QC samples or RSD > 30% in the QC samples were removed.^40,41^

### 2.8 Statistical analysis

The data of viable cell numbers, pH value and organic acids were expressed as mean ± SD, while one-way ANOVA analysis of the data was performed using SPSS 13.0 software. The data were tested for homogeneity of variance, and if the variances were found to be homogeneous, the Duncan method was used to compare the means between groups, otherwise the Games-Howell method was used to compare the means between groups. Differences were considered significant when *p* < 0.05.

The data analysis of metabolites was as follows, 798 peaks were detected and 741 metabolites were left after relative standard deviation de-noising. Then, the missing values were filled up by half of the minimum value. Also, internal standard normalization method was employed in this data analysis. The final dataset containing the information of peak number, sample name and normalized peak area was imported to SIMCA15.0.2 software package (Sartorius Stedim Data Analytics AB, Umea, Sweden) for multivariate analysis. Data was scaled and logarithmic transformed to minimize the impact of both noise and high variance of the variables. After these transformations, principle component analysis (PCA), an unsupervised analysis that reduces the dimension of the data, was carried out to visualize the distribution and the grouping of the samples. 95% confidence interval in the PCA score plot was used as the threshold to identify potential outliers in the dataset. Next, in order to visualize group separation and find significantly changed metabolites, supervised orthogonal projections to latent structures discriminate analysis (OPLS-DA) was applied. Then, a 7-fold cross validation was performed to calculate the value of *R*^2^*Y* and *Q*^2^. Afterwards, the parameters of three comparisons were *R*^2^*Y* = 1, 1, 0.998 and *Q*^2^ = 0.918, 0.975, 0.936 which were stable and good to fitness and prediction. Finally, 200 times permutations were further conducted. Here, the intercept value of *Q*^2^ = −0.13, −0.33, −0.09 represents the model had good robustness and no over-fitting phenomenon. Furthermore, the value of variable importance in the projection (VIP) of the first principal component in OPLS-DA analysis was obtained. It summarizes the contribution of each variable to the model. The metabolites with VIP > 1 and *p* < 0.05 (Student's *T* test) were considered as significantly changed metabolites, enabling the comprehensive analysis of the pathways of different metabolites (including enrichment analysis and topology analysis). After further screening, the primary metabolic pathway displaying the highest correlation with the different metabolites can be determined.^42^ In addition, commercial databases including KEGG (http://www.genome.jp/kegg/) and MetaboAnalyst (http://www.metaboanalyst.ca/) were used for pathway enrichment analysis.^43^

## 3. Results and discussion

### 3.1 Molecular weight distribution analysis

**Fig. 2:**
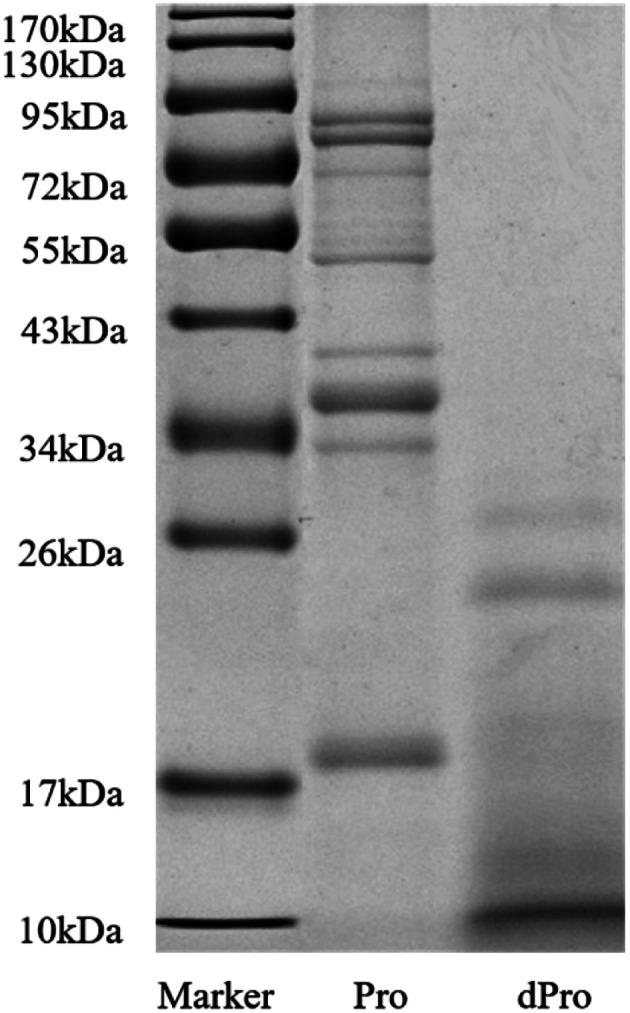
SDS-PAGE spectra of Pro and dPro. Marker = protein molecular weight standard (molecular weight from 10 to 170 kDa), Pro = soybean protein, dPro = digested soybean protein.

Fig. 2 and Table 3 show the molecular weight distribution of Pep, dPep, Pro, and dPro, respectively. Result in Fig. 2 indicated that after digestion *in vitro*, protein became peptides with a small molecular weight. All the peptides were concentrated below 34 kDa, and most of them were concentrated below 17 kDa. Table 3 shows that after digestion, the peptides with the small molecular weight increased. The total peptides were concentrated below 3000 Da, and the proportion of peptides < 1000 Da increased from 82.4% to 89.2%, while the proportion of peptides < 500 Da increased from 53.6% to 67.6%.

**Table 3:** Molecular weight distribution of Pep and dPep^a^

Molecular weight range (Da)	Pep		dPep	
	Integral area (%)	Comprehensive ratio (%)	Integral area (%)	Comprehensive ratio (%)
>3000	1.9	1.9	1.5	1.5
1500–3000	7.8	7.8	4.5	4.5
1000–1500	7.9	7.9	4.8	4.8
500–1000	28.8	82.4	21.6	89.2
<500	53.6		67.6	

a Pep = soybean oligopeptide, dPep = digested soybean oligopeptide.

### 3.2 The evaluation of the growth activity of *Lactobacillus acidophilus* JCM 1132

**Fig. 3:**
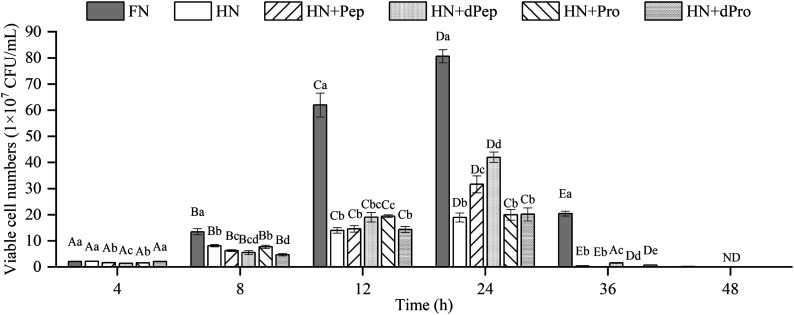
Viable cell numbers (1 × 10^7^ CFU mL^−1^) of *Lactobacillus acidophilus* JCM 1132 incubated for 48 h in different nitrogen source media. FN = MRS, HN = MRS without half nitrogen source, HN + Pep = HN with soybean oligopeptide, HN + dPep = HN with digested soybean oligopeptide, HN + Pro = HN with soybean protein, HN + dPro = HN with digested soybean protein. ND = not detection. Values are presented as mean ± SD, *n* = 3. Different capital letters indicate values in the same group at different time points with significant difference (*p* < 0.05). Different lowercase letters indicate values in six groups at each time point with significant difference (*p* < 0.05).

The change in viable cell numbers of *Lactobacillus acidophilus* JCM 1132 cultured for 48 h in different nitrogen source media is depicted in Fig. 3, indicating that during the 48 h cultivation period, the FN group maintained a relatively high number of viable cells. These results were significantly higher than in the other five groups (*p* < 0.05), indicating that Pep and Pro supplemented with equal amounts of nitrogen promoted bacterial growth, but the removed peptone, beef extract and yeast extract are rich in nitrogen sources, while also containing additional nutrients such as minerals, vitamins, and sugars. Therefore, the growth-promoting effect of the other groups was lower than the FN group.^44^

No significant difference was evident between the viable cell numbers of each group at 4 h (*p* > 0.05), while the FN group was significantly higher than the other groups during the 8–12 h period (*p* < 0.05), and no significant differences were apparent between the other groups (*p* > 0.05). However, the number of viable cells in the HN + Pep group reached 31.7 × 10^7^ CFU mL^−1^ at 24 h, which was significantly higher than that in the HN + Pro group (*p* < 0.05). For the digested samples, the viable cell numbers in the HN + dPep group reached 42.0 × 10^7^ CFU mL^−1^, which was significantly higher than the HN + dPro group (*p* < 0.05). Therefore, the results before and after digestion of the samples showed that the viable cell numbers in the Pep group were significantly higher than that in the Pro group (*p* < 0.05), indicating that Pep can better promote the growth of *Lactobacillus acidophilus* JCM 1132.

Comparing the Pep before and after digestion, the viable cell numbers in the HN + dPep group was significantly higher than that in the HN + Pep group at 24 h (*p* < 0.05), indicating that the growth-promoting effect of dPep surpasses that of Pep. Based on the analysis of the molecular weight distribution results, the molecular weight of Pep was smaller than Pro, while Pep was smaller than dPep. Therefore, the *Lactobacillus acidophilus* JCM 1132 was able to transport small peptides more efficiently as nutrients for growth. This result indicated that the probiotic effect of Pep, like other biological activities, is enhanced with small-molecule peptides.^45^ Comparing the Pro before and after digestion, at 8–12 h, the viable cell numbers in the HN + Pro group was significantly higher than in the HN + dPro group (*p* < 0.05). After Pro was digested *in vitro*, the hydrophobic inner core of the protein and long-chain peptides were produced, it is particularly easy for them to adhere to the cell membrane, forming pores to inactivate the cells.^46,47^ However, at 24 h there was no significant difference between HN + Pro group and HN + dPro group (*p* > 0.05), indicating that with the action of CEP, the HN + dPro group produced a certain amount of highly hydrophobic inner core of the protein and long-chain peptides. As for Pep and dPep, the Pep used in this study is a low-molecular-weight peptide produced by filtration through a composite membrane. The composite membrane removed the hydrophobic inner core of the protein and long-chain peptides mentioned above. So compare to Pro and dPro, Pep and dPep significantly increased the viable cell numbers and acid-producing ability of *Lactobacillus acidophilus* JCM 1132 (*p* < 0.05).

At 36 h, the HN + dPep group still maintained high viable cell numbers, and was significantly different from the HN + Pep group, the HN + Pro group, and the HN + dPro group (*p* < 0.05), indicating that Pep can maintain the viable cell numbers of *Lactobacillus acidophilus* JCM 1132 at a relatively high level, and prolong its growth time. Other studies also found that the viable cell numbers were prolonged after the addition of soybean, and that yogurt with peptides and soybean exhibited longer probiotic viability.^48–50^

### 3.3 The evaluation of the metabolic activity of *Lactobacillus acidophilus* JCM 1132

**Fig. 4:**
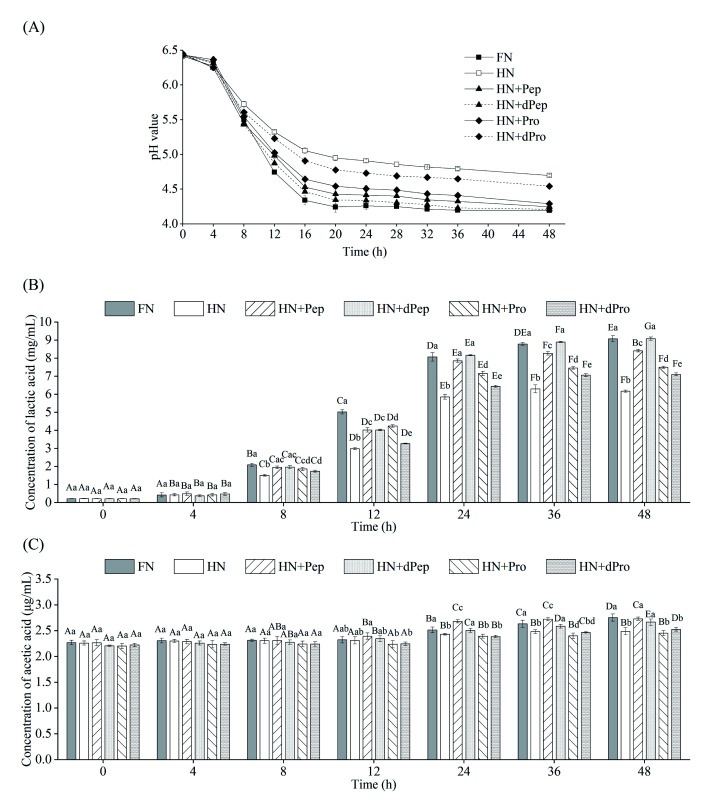
(A) pH (B) lactic acid (C) acetic acid produced by *Lactobacillus acidophilus* JCM 1132 incubated for 48 h in different nitrogen source media. FN = MRS, HN = MRS without half nitrogen source, HN + Pep = HN with soybean oligopeptide, HN + dPep = HN with digested soybean oligopeptide, HN + Pro = HN with soybean protein, HN + dPro = HN with digested soybean protein. Values are presented as mean ± SD, *n* = 3. Different capital letters indicate values in the same group at different time points with significant difference (*p* < 0.05). Different lowercase letters indicate values in six groups at each time point with significant difference (*p* < 0.05).

Fig. 4(A) shows the pH value changes in *Lactobacillus acidophilus* JCM 1132 cultured for 48 h in different nitrogen source media. These results indicated that 0–4 h was the delay period, 4–16 h was the logarithmic growth period, while the stable period was achieved after 16 h. The pH value of the bacterial culture broth of the different groups decreased with time, indicating that *Lactobacillus acidophilus* JCM 1132 produced metabolites and gradually reduced the pH value levels. Consequently, the pH value of the FN group decreased the most, from 6.4 to 4.2, followed by the HN + dPep group, the HN + Pep group, the HN + Pro group, and the HN + dPro group, while the smallest decrease was apparent in the HN group (*p* < 0.05). No significant differences were evident in any of the groups at 4 h (*p* > 0.05), while the pH value of the FN group significantly decreased after 8 h compared with the other groups (*p* < 0.05). However, after 24 h, the pH value of the HN + Pep group was significantly lower than that of the HN + Pro group (*p* < 0.05), the pH value of the HN + dPep group was significantly lower than that of the HN + dPro group (*p* < 0.05). Furthermore, the HN + Pep group and the HN + dPep group reached the same pH value level as the FN group (*p* > 0.05). The results showed that Pep promoted the acid production capacity of *Lactobacillus acidophilus* JCM 1132 significantly higher than Pro (*p* < 0.05), and the dPep is better than Pep.

The organic acid content in the culture media of different nitrogen source groups were determined to further evaluate the effect of Pep on the metabolic activity of *Lactobacillus acidophilus* JCM 1132, which is a homolactic fermentation, meaning that glucose undergoes glycolysis to produce pyruvate, and pyruvate only produces two molecules of lactic acid.^51^ However, some studies showed that *Lactobacillus acidophilus* JCM 1132 not only produces a large amount of lactic acid but also a small amount of acetic acid.^52^ Therefore, this study combined these findings to detect lactic acid and acetic acid content. Fig. 4(B) and (C) show the changes in the lactic acid and acetic acid content of *Lactobacillus acidophilus* JCM 1132 cultured in different nitrogen source media for 48 h. The lactic acid and acetic acid content increased with time, indicating that these substances were metabolized by *Lactobacillus acidophilus* JCM 1132. Fig. 4(B) shows that no significant differences were evident in the lactic acid content of any groups between 0–4 h (*p* > 0.05). During the 8–12 h period, the lactic acid content in the FN group was significantly higher than in the other groups (*p* < 0.05), the HN group was significantly lower than in the other groups (*p* < 0.05), while the differences between the remaining four groups were not significant (*p* > 0.05). However, after 24 h, the lactic acid content in the HN + Pep group reached 7.9 mg mL^−1^, which was significantly higher than that in the HN + Pro group (*p* < 0.05), while the lactic acid content in the HN + dPep group was 8.2 mg mL^−1^, which was significantly higher than that in the HN + dPro group (*p* < 0.05) and reached a level equivalent to the FN group (*p* > 0.05). In addition, the lactic acid content in the HN + dPep group was significantly higher than that in the HN + Pep group (*p* < 0.05), while samples subjected to the 36 h and 48 h periods followed this pattern as well. The results showed that the ability of Pep to promote *Lactobacillus acidophilus* JCM 1132 metabolism was significantly higher than Pro and dPep was substantially higher than Pep (*p* < 0.05).

Comparing the HN + Pro group and HN + dPro group, the pH value in the HN + Pro group was significantly lower than in the HN + dPro group during 12–48 h (*p* < 0.05) and the lactic acid content in the HN + Pro group was significantly higher than in the HN + dPro group during 12–48 h (*p* < 0.05). This result is similar to the viable cell number. It shows that the hydrophobic inner core of the protein and long-chain peptides in dPro inactivate the cells, resulting in the ability of dPro to promote the growth and metabolism of *Lactobacillus acidophilus* JCM 1132 significantly lower than Pro (*p* < 0.05).^46,47^

As for acetic acid, its initial concentration was 2.3 μg mL^−1^. Fig. 4(C) indicates that no significant differences were evident in the acetic acid content of each group during the 0–8 h period (*p* > 0.05), and could be ascribed to the addition of sodium acetate, as well as less acetic acid produced by *Lactobacillus acidophilus* JCM 1132.^53,54^ At 12 h, the acetic acid content of the HN + Pep group was higher than in the other groups, but the difference was not significant (*p* > 0.05). At 24 h, the acetic acid content of the HN + Pep group reached 2.7 μg mL^−1^, which was significantly higher than in the other groups (*p* < 0.05), while the acetic acid content in the HN + dPep group was significantly higher than in the HN + dPro group (*p* < 0.05). However, unlike the lactic acid result, although the HN + dPep group exhibited the same level of acetic acid as the FN group (*p* > 0.05), it was significantly lower than in the HN + Pep group (*p* < 0.05) and continued to follow this trend during the 36 h and 48 h periods. The results showed that the ability of Pep to promote the metabolism of *Lactobacillus acidophilus* JCM 1132 in producing acetic acid was significantly higher than Pro (*p* < 0.05). Furthermore, the findings involving the organic acids corresponded to the pH value results, indicating that *Lactobacillus acidophilus* JCM 1132 could reduce the pH value of the bacterial culture medium by producing lactic acid and acetic acid. These organic acids are beneficial metabolites produced by probiotics *via* glucose metabolism and are essential in inhibiting the growth of harmful microorganisms. Low pH value renders organic acids fat-soluble, which allows it to penetrate the cell membrane and reach the cytoplasm of pathogens, inhibiting their growth and improving the composition of intestinal flora, while providing health benefits for the host.^55–57^ Pep can promote *Lactobacillus acidophilus* JCM 1132 to produce lactic acid, acetic acid and significantly improving its metabolic capacity (*p* < 0.05). Therefore, this process may reveal the regulatory effect of Pep as a nitrogen source on probiotics, providing a theoretical basis for Pep to promote probiotic metabolism, as well as the changes in the composition of intestinal flora.^58^

An interesting phenomenon was revealed by combining the results of viable cell numbers and organic acids. The viable cell numbers of Pep before and after digestion were lower than that of the FN group (*p* < 0.05), but the content of lactic acid reached the same level as that of the FN group at 24 h (*p* > 0.05). Therefore, the viable cell numbers were not directly proportional to the organic acid content, indicating that Pep induced a more substantial improvement in the metabolic capacity of *Lactobacillus acidophilus* JCM 1132 than the capacity to promote growth. Some studies have shown that the growth rate of *Lactobacillus acidophilus* can be improved by adding whey peptide to the MRS medium, while the production of lactic acid and acetic acid also can be enhanced. However, whey peptide had a higher impact on metabolic capacity than growth capacity.^59,60^

### 3.4 Metabolite analysis

According to the results of viable cell numbers and organic acids, at 24 h, for the samples before and after digestion, Pep significantly enhanced the growth and metabolism ability of *Lactobacillus acidophilus* JCM 1132 compared with Pro (*p* < 0.05). Additionally, the viable cell numbers in the HN + dPep group were lower than in the FN group (*p* < 0.05), but the organic acid content reached the same level (*p* > 0.05). Consequently, to further explore the effect of the Pep on the metabolism of *Lactobacillus acidophilus* JCM 1132 and the difference between it and other samples in promoting metabolism, four groups (FN, HN, HN + dPep and HN + dPro) were selected for extraction and analysis of the metabolites in the different nitrogen source culture media at 24 h.

**Fig. 5:**
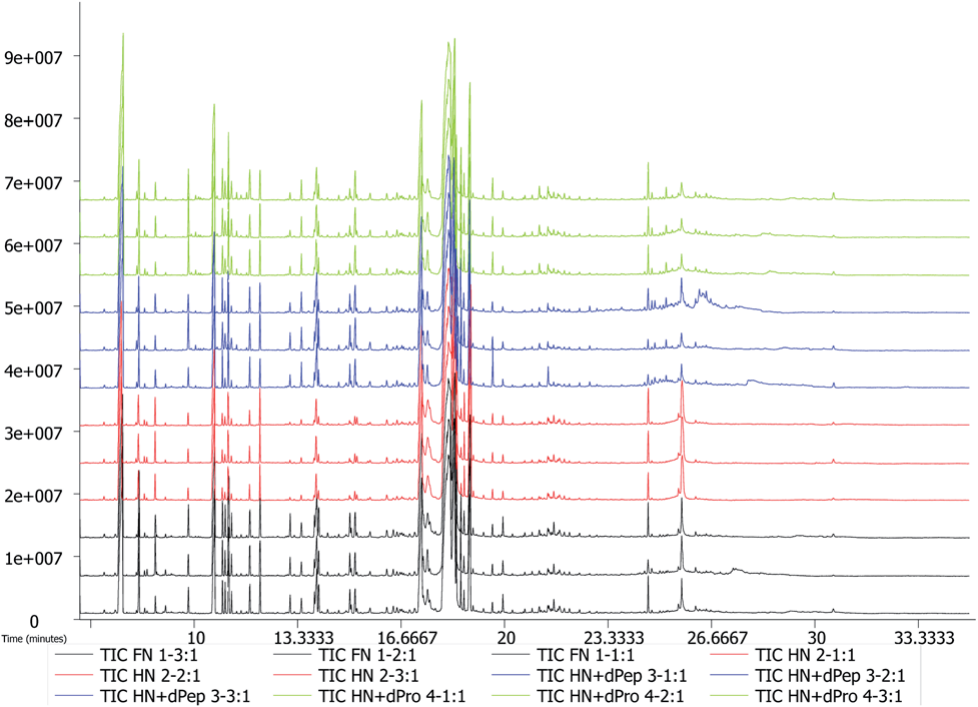
GC-TOF-MS total ion chromatogram (TIC) of *Lactobacillus acidophilus* JCM 1132 in different nitrogen source media. FN = MRS, HN = MRS without half nitrogen source, HN + dPep = HN with digested soybean oligopeptide, HN + dPro = HN with digested soybean protein.

**Fig. 6:**
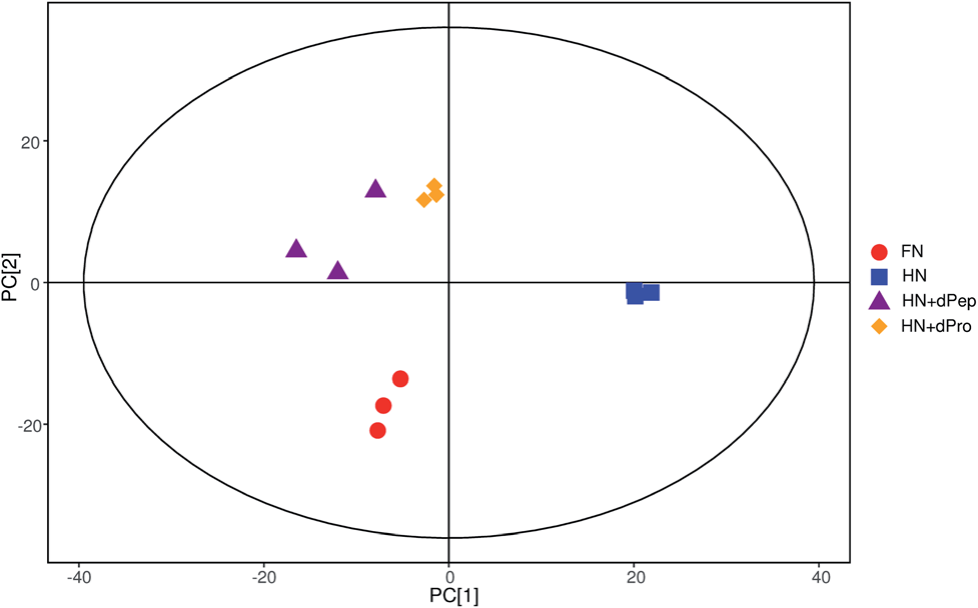
Score scatter plot for PCA model of metabolites cultured in different nitrogen source media. The *x*-axis PC [1] and *y*-axis PC [2] represents the aggregation of the first and second principal components, respectively. The scatter color and shape represent the experimental grouping of the samples. The samples are all within the 95% confidence interval (Hotelling's *T*-squared ellipse). FN = MRS, HN = MRS without half nitrogen source, HN + dPep = HN with digested soybean oligopeptide, HN + dPro = HN with digested soybean protein.

Fig. 5 presents the total ion chromatogram obtained *via* GC-TOF-MS in different nitrogen source culture media at 24 h. The results indicated that 798 peaks were detected in the *Lactobacillus acidophilus* JCM 1132 culture media, while 258 annotated metabolites were identified, which included about 62 organic acids and their derivatives, 39 amino acid peptides, 32 carbohydrates and their conjugates, 24 alcohols, 17 amines and 9 fatty acids and their conjugates. The PCA results showed that metabolites were noticeably separated among four groups and the three samples in each group had good parallelism (Fig. 6).^44,61^

**Fig. 7:**
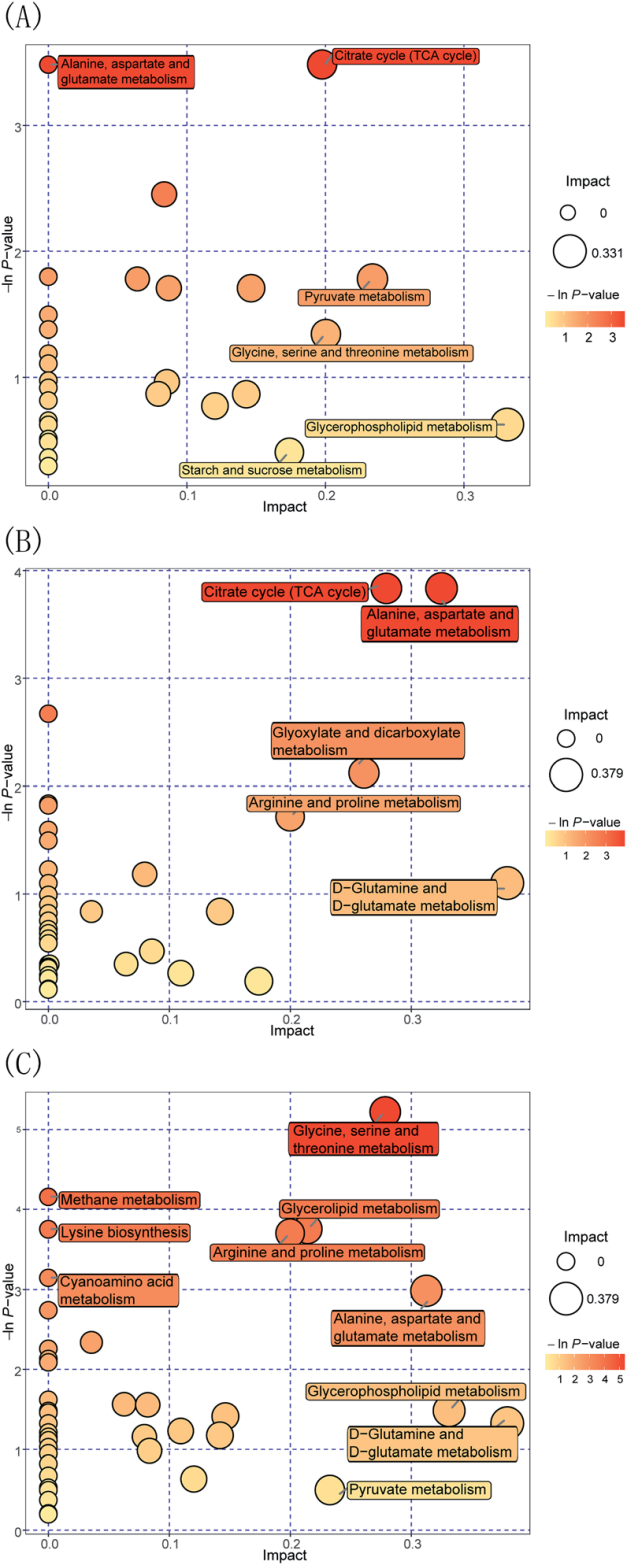
Metabolome view maps of the common metabolites identified in different nitrogen source media. (A) HN + dPep *vs.* FN (B) HN + dPep *vs.* HN (C) HN + dPep *vs.* HN + dPro. The *x*-axis represents the pathway impact, and *y*-axis represents the pathway enrichment. Larger sizes and darker colors represent higher pathway enrichment and higher pathway impact values. FN = MRS, HN = MRS without half nitrogen source, HN + dPep = HN with digested soybean oligopeptide, HN + dPro = HN with digested soybean protein.

Furthermore, to evaluate the way in which Pep enhances the *Lactobacillus acidophilus* JCM 1132 metabolism compared to the MRS medium, the FN group was compared to the HN + dPep group. Fig. 7(A) shows the metabolic pathway analysis of the 24 h culture media with the FN group and the HN + dPep group, and it was found that 31 metabolic pathways were enriched (*p* < 0.05, VIP > 0.1). Of these, alanine, aspartate, and glutamate metabolism, the TCA cycle, the pyruvate metabolism, glycine, serine, and threonine metabolism, glycerophospholipid metabolism, as well as starch and sucrose metabolism are significantly correlated with differential metabolites. The dPep significantly increased the oxaloacetic acid content (*p* < 0.05, VIP > 0.1), while the pyruvic acid content was significantly reduced (*p* < 0.05, VIP > 0.1). All of these are intermediate products of the metabolic pathways such as alanine, aspartic acid and glutamic acid metabolism, the TCA cycle, and sulfate acid metabolism. During sugar metabolism, pyruvate can be converted into acetyl-CoA and oxaloacetic acid to enter the TCA cycle, indicating that dPep promotes the conversion of pyruvic acid to oxaloacetic acid and enhances the TCA cycle, thereby enhancing the *Lactobacillus acidophilus* JCM 1132 metabolism (Table 4).^62^

**Table 4:** Metabolic pathways enrichment from the significantly different metabolites in different nitrogen source media^a^

Comparison	Pathway	Hits	Hit differential metabolites	
			Up	Down
A: HN + dPep *vs* FN	TCA cycle	3	Oxaloacetic acid	Pyruvic acid
				Succinic acid
	Alanine, aspartate and glutamate metabolism	3	Oxaloacetic acid	Pyruvic acid
				Succinic acid
B: HN + dPep *vs* HN	TCA cycle	4	Oxoglutaric acid	—
			Citric acid	
			Oxaloacetic acid	
			Fumaric acid	
	Alanine, aspartate and glutamate metabolism	4	l-Aspartic acid	—
			Oxoglutaric acid	
			Oxaloacetic acid	
			Fumaric acid	
C: HN + dPep *vs* HN + dPro	Glycerophospholipid metabolism	2	—	Glycerol 3-phosphate
				Ethanolamine

a FN = MRS, HN = MRS without half nitrogen source, HN + dPep = HN with digested soybean oligopeptide, HN + dPro = HN with digested soybean protein.

Moreover, to evaluate the way in which Pep was used as a nitrogen source to enhance the metabolism of *Lactobacillus acidophilus* JCM 1132, Fig. 7(B) shows the metabolic pathway analysis of the 24 h culture media with the HN + dPep group and the HN group, 38 metabolic pathways were enriched (*p* < 0.05, VIP > 0.1). Of these, the TCA cycle, alanine, aspartate, and glutamate metabolism, arginine and proline metabolism, glyoxylate and dicarboxylate metabolism, as well as the d-glutamine and d-glutamate metabolism are significantly correlated with differential metabolites. Regarding sugar metabolism, dPep significantly increased the content of the oxoglutaric acid, the citric acid, the oxaloacetic acid, and the fumaric acid (*p* < 0.05, VIP > 0.1), which are all intermediates of the TCA cycle and are essential substances for energy production. During amino acid metabolism, dPep significantly increased the content of l-aspartic acid (*p* < 0.05, VIP > 0.1), a sugar-forming amino acid that could be converted into oxaloacetic acid to enter the TCA cycle. The content of both l-aspartic acid and oxaloacetic acid were increased (*p* < 0.05, VIP > 0.1), indicating that the dPep enhanced the metabolism of *Lactobacillus acidophilus* JCM 1132 by promoting the metabolism of alanine, aspartic acid, glutamic acid, and the TCA cycle (Table 4).^63^

Additionally, to evaluate the way in which Pep enhanced the metabolism of *Lactobacillus acidophilus* JCM 1132 compared to Pro, the HN + dPep group was compared with the HN + dPro group, Fig. 7(C) shows the metabolic pathway analysis of the 24 h culture media with the HN + dPep group and the HN + dPro group, 43 metabolic pathways were enriched (*p* < 0.05, VIP > 0.1). Of these, the glycine, serine, and threonine metabolism, the arginine and proline metabolism, the methane metabolism, lysine biosynthesis, alanine, aspartate and glutamate metabolism, cyanoamino acid metabolism, glycerol phospholipid metabolism, d-glutamine, and d-glutamate metabolism, as well as the pyruvate metabolism are significantly correlated with differential metabolites. Among all pathways, amino acid-related metabolic pathways are abundant, suggesting that dPep significantly increased the amino acid metabolism of *Lactobacillus acidophilus* JCM 1132 by significantly upregulating amino acids such as l-aspartic acid and l-homoserine (*p* < 0.05, VIP > 0.1). Glycerol phospholipids represents the most abundant type of phospholipids in the body, which are essential during phospholipid metabolism, while being the main components in biofilms, and participates in the process of protein recognition and signal transduction in cell membranes. Both glycerol 3-phosphate and ethanolamine are essential intermediates for glycerol phospholipid metabolism. The former can be obtained by converting the glycolysis intermediate dihydroxyacetone phosphate, while the latter is a precursor of brain phospholipid synthesis. The dPep significantly reduced glycerol phospholipid synthesis by downregulating the glycerol 3-phosphate and ethanolamine content (*p* < 0.05, VIP > 0.1), and the results of growth and metabolism showed that dPep significantly promoted the growth and metabolism of *Lactobacillus acidophilus* JCM 1132 than dPro (*p* < 0.05). Combining the two results, the hydrophobic inner core of the protein and long-chain peptides in dPro destroyed the cell membrane of *Lactobacillus acidophilus* JCM 1132, the components of cell membrane were decomposed, and the intermediate products of glycerol phospholipid metabolism were piled up in large quantities, and eventually it leads to cell inactivation. However, dPep was directly transported into the cell, and the cell membrane was not damaged. The intermediate products of glycerol phospholipid metabolism were transformed into the components of cell membrane (Table 4).^64,65^

## 4. Conclusions

In this study, Pep and Pro are digested *in vitro*, and the media are prepared by equal nitrogen replacement. The effect of Pep on the growth and metabolism of *Lactobacillus acidophilus* JCM 1132 is explored by comparing the differences between Pep and other groups. The results indicate that the ability of Pep in promoting the growth of *Lactobacillus acidophilus* JCM 1132 is lower than the MRS medium (*p* < 0.05). However, by influencing its metabolic pathway, pyruvate is converted into oxaloacetic acid and lactate, significantly increasing the l-aspartic acid content, while enhancing the TCA cycle and the amino acid metabolism, consequently, substantially improving its metabolic ability (*p* < 0.05, VIP > 0.1). Additionally, the ability of dPep in promoting the growth and metabolism of *Lactobacillus acidophilus* JCM 1132 is higher than the dPro (*p* < 0.05). Due to the low molecular weight of dPep, it can be better transported and utilized. And dPep significantly strengthen the amino acid metabolism by upregulating the l-aspartic acid and weakened the metabolism of glycerol phospholipid by downregulating 3-phosphoglycerol (*p* < 0.05, VIP > 0.1). However, the absorption of Pep in the small intestine needs to be considered in subsequent experiments, which will lead to the failure of Pep to act on the intestinal flora, and the specific content needs further verification *in vivo* study.

## Conflicts of interest

There are no conflicts to declare.

## Abbreviations

Pep: Soybean oligopeptide
dPep: Digested soybean oligopeptide
Pro: Soybean protein
dPro: Digested soybean protein
HN: MRS medium without half nitrogen source
HN + Pep: HN supplemented Pep
HN + dPep: HN supplemented dPep
HN + Pro: HN supplemented Pro
HN + dPro: HN supplemented dPro
TCA: Tricarboxylic acid
BCA: Bicinchoninic acid
SDS: Sodium dodecyl sulfate
CEP: Cell-envelope proteinase
TEMED: Tetramethylethylenediamine
BSTFA: Bis trifluoroacetamide
FAME: Fatty acid methyl ester
TMCS: Trimethyl chlorosilane
TFA: Trifluoroacetic acid
SDS-PAGE: Sodium dodecyl sulfate-polyacrylamide gel electrophoresis
HPLC: High performance liquid chromatography
DAD: Diode array detector
QC: Quality control
ANOVA: Analysis of variance
PCA: Principal component analysis
OPLS-DA: Orthogonal projections to latent structures discriminate analysis
VIP: Variable importance in the projection

## Supplementary Material
